# Cervical lymph node metastasis as the first symptom of combined anaplastic thyroid carcinoma (squamous cell carcinoma) and follicular carcinoma: a case report

**DOI:** 10.1186/s12902-024-01617-1

**Published:** 2024-06-12

**Authors:** Jiazhen Li, Zhijun Ma, Deshou Ma, Yusufu Maimaiti, Shuyun Jiang, Xiaowu Wang

**Affiliations:** 1 Department of Surgical Oncology, The Affiliated Hospital of Qinghai University, Xining, Qinghai Province China; 2 Department of Clinical Medicine, Qinghai University, Xining, Qinghai Province China; 3 Department of Head and Neck Surgery, Hubei Cancer Hospital, Wuhan, Hubei Province China

**Keywords:** Primary squamous cell carcinoma of the thyroid, Anaplastic thyroid carcinoma, Follicular thyroid carcinoma, Lymph node, Metastasis

## Abstract

**Background:**

Anaplastic thyroid carcinoma(ATC) is a rare pathological type of thyroid malignancy. Primary squamous cell carcinoma of thyroid(PSCCT) is now considered as a subtype of ATC, hereinafter referred to as ATC-SCC subtype. ATC-SCC subtype combined with follicular thyroid carcinoma is exceedingly rare, with fewer cases reported. The ATC-SCC subtype is a highly invasive tumor with a poor prognosis for patients after metastasis occurs, and current treatment of this type of tumor is tricky.

**Case presentation:**

A 68-year-old female patient presented with a gradually growing swelling of right cervical region. Comprehensive auxiliary examinations and postoperative pathology confirmed the diagnosis of ATC-SCC subtype with follicular thyroid carcinoma, and the metastasis squamous cell carcinoma of the right cervical lymph nodes originates from ATC-SCC subtype. The patient received chemoradiotherapy postoperative. However, the residual cervical lymph nodes metastasis with squamous cell carcinoma still infiltrated surrounding structures in the neck extensively after palliative resection. The patient died 7 months after surgery.

**Conclusion:**

Our case highlights that cervical lymph node metastasis may be a significant factor in the poor prognosis of ATC-SCC subtype. This malignancy should be detected and treated early.

## Background

PSCCT has been classified as a subtype of ATC according to the fifth edition of the WHO New Classification of Endocrine and Neuroendocrine Tumors.Prior to this, scholars generally regarded PSCCT as a separate malignancy. ATC-SCC subtype is a rapidly growing, highly aggressive malignant tumour of epithelial tissue that occurs in the elderly and accounts for less than 1% of all thyroid malignancies, although the etiology is unclear [1]. Simultaneous malignancies with multiple histological origins, including ATC-SCC subtype, in the thyroid are rare, and the diagnosis and treatment of such multicomponent tumors is currently very challenging [2]. To the best of our knowledge, there are no reported cases of ATC-SCC subtype combined with follicular thyroid carcinoma in which metastatic squamous carcinoma of the lymph nodes in the lateral cervical region was first detected, now we report one case.

## Case presentation

**Fig. 1:**
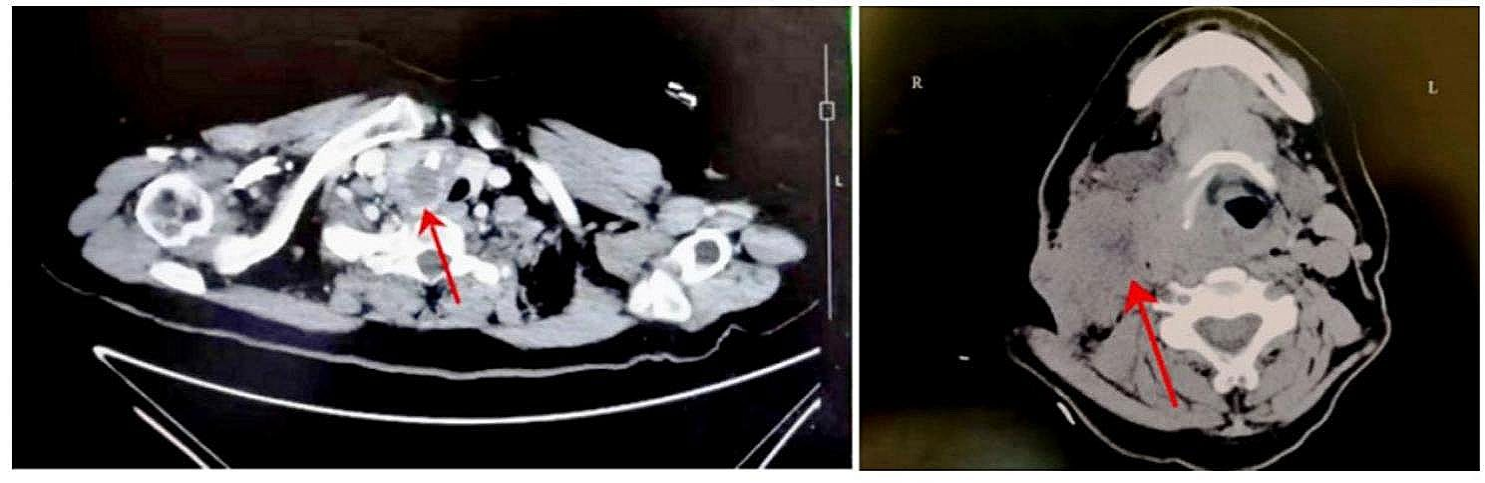
Enhanced CT of the thyroid gland. This picture suggests a malignant tumor right lobe of thyroid gland, with multiple enlarged and fused lymph nodes in the right cervical space

A 68-year-old female patient was admitted to the Affiliated Hospital of Qinghai University due to the gradual enlargement of the right cervical mass for 3 months. No complaints of pain in the neck mass, hoarseness, difficulty breathing or swallowing. No history of radiation to the neck or family history of thyroid carcinoma. No iodine deficiency. Physical examination: We touched a palpable 2 × 2 cm mass on the right thyroid gland, firm, non-tender, unclear boundary, and moving up and down with swallowing. We also found a palpable 3 × 2 cm mass in the right cervical region with hard quality, no tenderness, unclear boundary and poor motion. Thyroid function is normal (T3, T4, TSH, TPOAb and Tg). Cervical ultrasound showed that a 2.1 × 2.0 cm heterogenous echoic nodules were seen in the right lobe of the thyroid gland (TI-RADS grade: 4a) and several hypoechoic nodules of different sizes were found in the left lobe of the thyroid gland, simultaneously there was an enlarged lymph node in right cervical region II about 3.1 × 2.6 cm, unclear boundary between the cortex and medulla, and disappearance of lymphatic hilum. Enhanced computed tomography(CT) of the thyroid suggests malignant thyroid occupancy in the right lobe, multiple small nodules in the left lobe, multiple enlarged lymph nodes in the right cervical space, some of which were fused to each other and measure approximately 3.32 × 3.14 cm (Fig. 1).

**Fig. 2:**
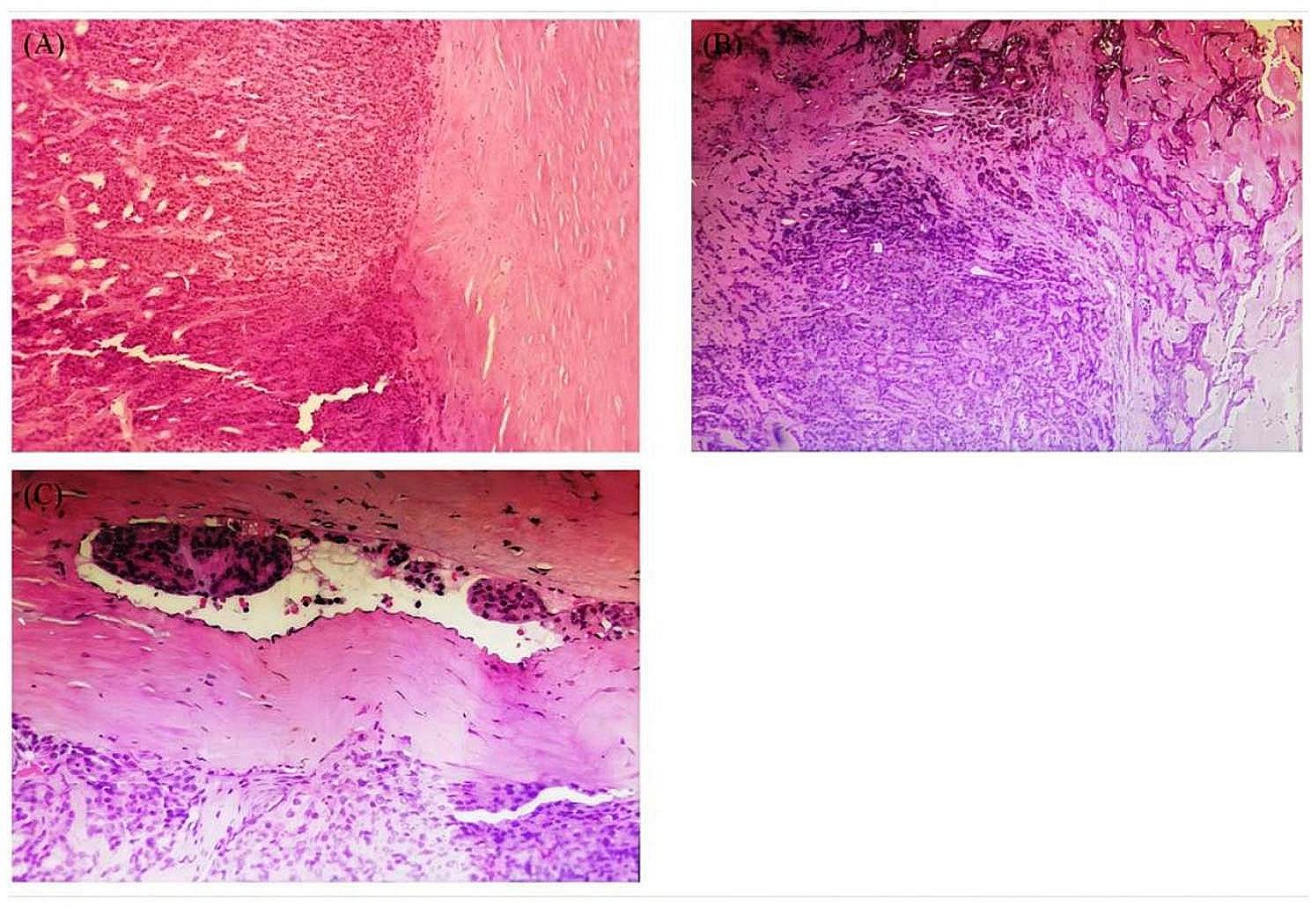
Postoperative pathology of right thyroid tumour tissue suggestive of squamous cell carcinoma of the thyroid gland with lymphatic vessel invasion. (hematoxylin-eosin stain, **A**:×4; **B**: ×10; **C**:×40)

The trachea mildly compressed and deviated to the left. Ultrasound-guided fine-needle aspiration of the right cervical region II lymph node suggests squamous carcinoma metastasis. Fine-needle aspiration cytology of the right thyroid lobe (multi-site sampling) confirmed follicular neoplasm. In addition to thyroid lesions, there were no suspicious primary squamous carcinomas lesion in head, neck or distant organs after comprehensive clinical, endoscopic and imaging examinations (CT of the chest, head, whole abdomen and nasopharynx as well as gastrointestinal endoscopes and laryngoscopy). Subsequent multidisciplinary consultation considered that the metastatic squamous carcinoma of the right cervical lymph node was still likely to be thyroid-derived. The patient underwent total thyroidectomy and partial right cervical lymph node dissection (levels III, IV, VB&VI) after perfect preoperative preparation. At surgery, we observed that a huge mass about 2 × 2 cm occupied almost the entire right thyroid lobe, breaking through the fibrous capsule thyroid gland and adhering to the surrounding muscle tissue, but there is no invasion of the recurrent laryngeal nerve and trachea. Gross specimens showed a greyish-white, tough, partially calcified interior on incision of the mass. When lymph node dissection was performed, we found that the lymph nodes in the right cervical region II were significantly enlarged and it severe adhesions with the internal jugular vein and common carotid artery, fusion of metastatic lymph nodes into a mass encircling the carotid artery making it difficult to achieve complete separation and total removal, therefore a palliative resection was carried out. The postoperative pathology report confirmed follicular carcinoma with moderately differentiated squamous cell carcinoma of the right thyroid gland, with lymphatic vessel invasion, nodular goiter on the left thyroid gland(Fig. 2).

**Fig. 3:**
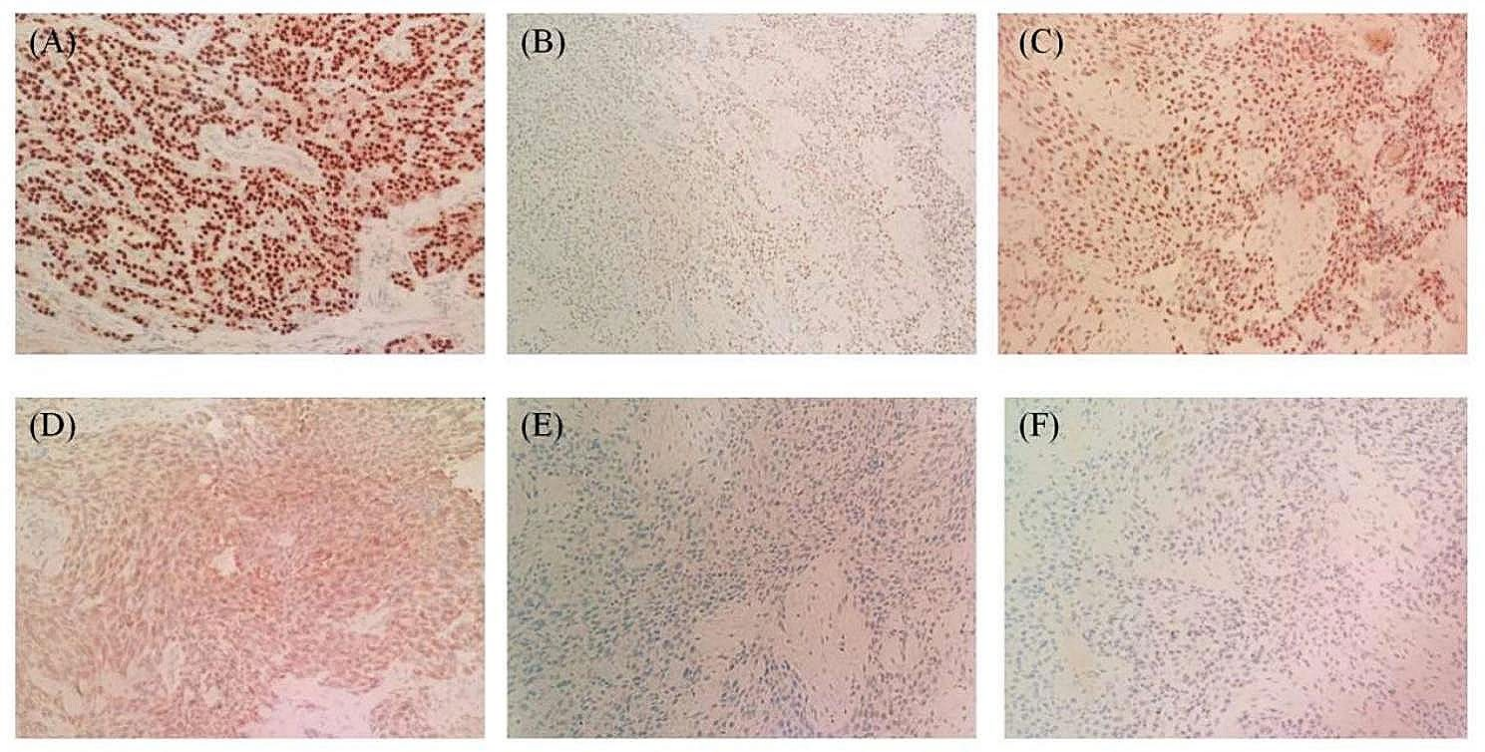
Immunohistochemical profile in the squamous carcinoma tissue area. **A** PAX-8:positive (×20), **B** P40:positive (×10), **C** P63:positive (×20), **D** CK-19:positive (×20), **E** Tg: negative(×20), **F** TTF-1:negative(×20)

There was no metastasis of the lymph nodes in the central region of the right neck. Of the 13 lymph nodes removed from the lateral neck region, 5 showed metastasis of squamous carcinoma. Immunohistochemical staining of the squamous cancer cells showed positive staining for PAX-8(Fig. 3A), P53, P40(Fig. 3B), P63(Fig. 3C), CEA, AE1/ AE3 and CK19(Fig. 3D), negative in thyroglobulin (Tg)(Fig. 3E), TTF-1(Fig. 3F) and Bcl-2, and the Ki-67 labeling index was 40%.BRAFV600E mutation negative. Based on postoperative histomorphological and immunohistochemical findings, we identified this patient as having ATC-SCC subtype in combined with follicular thyroid carcinoma, and metastatic squamous carcinoma of the lymph nodes in the neck which originated from ATC-SCC subtype. According to the 8th edition of the AJCC/TNM staging system for thyroid cancer, the ATC-SCC subtype is stage IVB.

Thyroid-stimulating hormone (TSH) suppressive therapy and calcium supplementation were given after operation. Further treatment plans include iodine-131 treatment for the follicular component of the thyroid tumor, and systemic chemotherapy combined with neck 3D intensity modulated radiation therapy for the ATC-SCC subtype. The patient completed 2 cycles (1/25d) of 5-fluorouracil(750 mg/m2 d1-5) combined with cisplatin(30 mg d1-3) chemotherapy followed by neck radiotherapy. The initial radiotherapy dose was 600 cGy/3f, with a gradual increase to a planned dose of 6000 cGy/30f at a later stage, but our patient experienced severe side-reactions such as nausea and vomiting after completing the 4th cycle of radiotherapy and was given a palliative radiotherapy dose of 5000 cGy/25f in the 5th cycle after consultation with the patient and her family. The patient abandoned subsequent iodine-131 treatment. Residual lymph node squamous carcinoma metastasis in region II of the cervical after palliative resection invaded the right neck tissue extensively with localized skin fester, combine with cachexia, the patient died 7 months after surgery.

## Discussion and conclusions

Based on the patient complaints and auxiliary examinations, we first identified metastasis squamous cell carcinoma of the right cervical lymph nodes and follicular thyroid neoplasm. The next major step was to pinpoint the primary lesion of squamous cell carcinoma, such as laryngeal, nasopharyngeal, respiratory and gastrointestinal tract malignancies that are prone to metastasis to the cervical lymph nodes. There were no primary squamous carcinoma lesions at the above sites after completion of ultrasound, CT, endoscopy and other ancillary examinations. Following the MDT meeting, patients were still not excluded from ATC-SCC subtype, but histopathology and immunohistochemistry are currently the surest way for diagnosis of PSCCT [3], surgery is recommended for diagnosis and treatment. Post-operative histopathology and immunohistochemistry confirmed that the patient had ATC-SCC subtype combined with follicular thyroid carcinoma and metastatic squamous carcinoma of the lymph nodes in the neck which ATC-SCC subtype originated.

Squamous epithelial cells absence in the normal thyroid gland. When squamous cell carcinoma of the thyroid gland is detected during the diagnostic, it should be differentiated from secondary squamous cell carcinoma of the thyroid gland. Occasional secondary squamous carcinomas of the thyroid gland often arise from direct infiltration or metastasis of squamous carcinomas of the respiratory or digestive tracts, the most common of which is laryngeal squamous cell carcinoma [4]. In this case, secondary squamous thyroid carcinoma has been ruled out in the search for the primary lesion of the metastasis of squamous carcinoma in the cervical lymph nodes. As a subtype of ATC, the clinical presentation and pathologic features of PSCCT share numerous similarities with ATC, for example, highly malignant and invasive are their characteristics. The major cellular subtypes of ATC include sarcomatoid, and epithelial (i.e., squamoid), pleomorphic giant cell; rarer variants include rhabdoid, osteoclastic, angiomatoid, etc. Some cases exhibit a mixture of cellular subtypes [5, 6]. For this case, in addition to follicular thyroid carcinoma, the tumor tissue microscopically showed only moderately differentiated squamous carcinoma cells, without other cellular subtypes were seen. Therefore, based on histopathology, we considered this patient as a possible ATC-SCC subtype. Immunohistochemistry stains may help confirm the diagnosis, AE1/ AE3 and PAX8 are commonly expressed positively in ATC, while Tg and TTF-1 are generally expressed negatively. And based on previous reports of PSCCT, CK19, P63, P40 positive staining are sensitive markers for the diagnosis of SCCT, P40 is more specific than P63 for the identification of squamous carcinoma [7]. In addition, the mitotic rate (Ki-67) is typically > 30%, our patient had a Ki-67 labeling index of 40%, indicating active tumor cell proliferation and also indicating a poor prognosis. Thus, the presence of ATC-SCC subtype is supported by our patient’s histological and immunohistochemical.

​In the early stages of ATC-SCC subtype, there may be no specific clinical features, but the tumor grows rapidly and is highly invasive, the symptoms such as hoarseness, dyspnea and dysphagia may appear in later stages, and the rate of lymph node and distance metastasis is significantly higher than that of differentiated thyroid cancer [8]. However, at the time of presentation our patient had no obvious clinical manifestation of compression of the trachea or esophagus, but by this time the squamous carcinoma had metastasised to the cervical lymph nodes and invaded vital vessels of the neck. The residual postoperative lymph node metastases invade the soft tissues of the neck extensively in a relatively short period of time, as evidenced by their highly biological aggressiveness. At present, the nosetiology of ATC-SCC subtype is unclear, there are three hypotheses about its origin [9]: (1) the “embryonic nest theory” suggests that the embryonic remnants of squamous epithelial cells (e.g. thymic epithelium, thyroglossal duct, etc.) evolve into squamous epithelial cells during development; (2) the “metaplasia theory” holds a view that chronic inflammatory conditions such as Hashimoto’s thyroiditis may lead to chemotaxis of the follicular epithelium of the thyroid to squamous epithelium; (3) at “dedifferentiation theory” assumes that papillary or follicular carcinoma of the thyroid may dedifferentiate to squamous cell carcinoma. In our case, it is possible that the patient had squamous cell carcinoma intermingled with follicular carcinoma in the thyroid gland over a long period of time, and from the thyroid function test report we can rule out Hashimoto’s thyroiditis, therefore we prefer the “dedifferentiation theory” above hypothesis. However, whether ATC-SCC subtype is derived from the dedifferentiation of FTC tumor cells still needs further investigation.

Combination therapy for each tumor component is the fundamental principle of treatment for mixed tumours [10]. Recommended according to NCCN guidelines (2022), the patient underwent a total thyroidectomy to remove the follicular carcinoma. Levothyroxine which follows, aimed at hormone replacement and TSH suppressive therapy. The patient was also advised to undergo further treatment with iodine-131. Multimodal therapy is currently recommended for ATC, including surgical resection (for lesions that can be resected), external radiation radiotherapy, chemotherapy, and/or targeted therapies [11]. The NCCN guidelines propose total thyroidectomy to achieve complete (R0)/microscopic residual (R1)/debulking or incomplete surgery (R2) for operable patients, and radiation should be considered early in the treatment of ATC. Chemotherapy regimens including taxanes (paclitaxel or docetaxel), platins (cisplatin or carboplatin), etc. In addition, early evaluation of tumor mutations to determine if a patient is a candidate for targeted therapy.

However, at the time of this patient’s visit to our hospital, PSCCT was not considered as a subtype of ATC, there were limited adjuvant treatment regimens available to us postoperatively. Therefore, according to previous reports of PSCCT treatment and chemotherapy regimens for squamous carcinoma of the head and neck, we took 5-fluorouracil in combination with cisplatin for treatment, followed by radiotherapy to the area of the neck invaded by the carcinoma. Currently, PSCCT is a subtype of ATC, and perhaps in the future such cases will be able to refer to treatment protocols for ATC.

At present, early detection of mutations in ATC patients and the administration of appropriate targeted therapy as soon as possible is positive for improving prognosis. BRAFV600E is the most common mutation in ATC and can be treated with dabrafenib/trametinib, but this patient was negative for the BRAFV600E mutation. Studies have shown that most ATC patients have the BRAFV600E mutation [12]; however, this patient was negative for the BRAFV600E mutation, we considered the possibility that this patient may have had additional ATC-SCC subtype drivers or that the tumor had acquired “late” driver mutation, such as TP53 or TERT [13].

Lenvatinib, a multi-target tyrosine kinase inhibitor (TKI), is recommended for the treatment of radioiodine-refractory thyroid cancer. A recent report of a patient with secondary squamous thyroid cancer with multiple regional lymph node metastases who was treated with lenvatinib following surgery showed a significant diameter reduction of remaining lymph nodes involved [14]. It hints that lenvatinib perhaps effective in the treatment of ATC-SCC subtype. The research has shown that PD-L1 is positively expressed in some patients with ATC, including the ATC-SCC subtype [15, 16], there are also studies suggested that immunotherapy may be an effective option for ATCs [17]. Unfortunately, the patient refused to try the above treatments for financial reasons.

​The patient’s prognosis normally depends on the complete removal of the lesion, and according to statistical analysis, the 3-year survival rate for patients with complete removal of the lesion was 43.1%, compared to 15.9% for patients with partial removal [18, 19]. In this case, the lymph nodes of the right cervical region II involved were severely adhered to the internal jugular vein and common carotid artery, despite our patient underwent chemotherapy and radiotherapy after cervical lymph nodes palliative resection, the prognosis remained poor, this leads us to conclude that the failure to achieve R0 resection of such tumors is a key factor in the poor prognosis. Nonetheless, some scholars have suggested that lymph node metastases do not affect the prognosis of PSCCT patients [20], conversely lymph node involvement in this patient happens to be one of the important factors for poor prognosis, so it is assumed that the prognostic impact of lymph node metastasis may be related to the area of metastasis, T-stage and the possibility of complete surgical resection.

​Preoperative diagnosis and treatment of ATC-SCC subtype in combination with follicular thyroid carcinoma is challenging, the difficulty of making a valid diagnosis with the common imageological examination and fine-needle aspiration cytology, histopathology and immunohistochemistry are currently the most reliable means of making a definitive diagnosis. Given the highly aggressive feature of ATC-SCC subtype, it should be treated as early as possible and complete surgical excision is crucial to the prognosis. Moreover, when cervical lymph node metastasis occurs in ATC-SCC subtype and the lesion cannot be completely removed, effective pre/postoperative targeted therapy may improve the patient’s prognosis.

## Data Availability

The datasets used and/or analysed during the current study available from the corresponding author on reasonable request.
